# The complete chloroplast genome sequence of *Thladiantha nudiflora* Hemsl. ex F.B.Forbes & Hemsl. 1887 (Cucurbitaceae)

**DOI:** 10.1080/23802359.2024.2305402

**Published:** 2024-01-25

**Authors:** Yan-Yan Zhao, Min-Min Chen, Bai-Lin Duan, Qing-Zhou Xie, Qiang Miao

**Affiliations:** a Plant Genomics and Molecular Improvement of Colored Fiber Laboratory, College of Life Sciences and Medicine, Zhejiang Sci-Tech University, Hangzhou, China; b Zhejiang Province Key Laboratory of Plant Secondary Metabolism and Regulation, College of Life Sciences and Medicine, Zhejiang Sci-Tech University, Hangzhou, China; c Fuyang Center for Agro-technical Popularization of Hangzhou, Hangzhou, China

**Keywords:** Cucurbitaceae, chloroplast genome, phylogeny, *Thladiantha nudiflora*

## Abstract

*Thladiantha nudiflora* Hemsl. ex F.B.Forbes & Hemsl. 1887 (Cucurbitaceae) has been widely known as a traditional medicine plant. In this study, we sequenced, assembled, and annotated the complete chloroplast genome of *T. nudiflora*. The chloroplast genome of *T. nudiflora* is 156,824 base pair (bp) in length, containing a large single-copy region of 86,566 bp and a small single-copy region of 18,070 bp, separated by a pair of inverted repeats of 26,094 bp. The chloroplast genome contains 132 genes, including 87 protein-coding, 37 transfer RNA, and eight ribosomal RNA genes. Phylogenetic analysis of the chloroplast genome revealed that species of the genus *Thladiantha* were clustered together in the phylogenetic trees. This study will not only shed light on *T. nudiflora*’s evolutionary position but also provide valuable chloroplast genomic information for future studies into the origins and diversification of the genus *Thladiantha* and the Cucurbitaceae family.

## Introduction

Cucurbitaceae is a family of climbers or trailers native to tropical and subtropical climates, with approximately 960 accepted species (Schaefer et al. 2009). *Thladiantha nudiflora* Hemsl. ex F.B.Forbes & Hemsl. 1887 is a species belonging to the genus *Thladiantha* in the Cucurbitaceae family. It is native to Eastern Asia (Anh et al. 2020). *Thladiantha nudiflora* prefers sunny and fertile conditions. It is distributed mainly in South and Southeast Asia (Xu and Le 2017). Traditional Chinese medicine has employed *Thladiantha* species to soften hard masses, clear heat and detoxify, reduce swelling, and get rid of carbuncles (Nie et al. 1989; Wang et al. 2011). The first complete *T. nudiflora* chloroplast genome was identified and described in this article. This study offers potential genetic data for Cucurbitaceae phylogenetic and systematic molecular ecology investigations.

## Materials and methods

**Figure 1.**
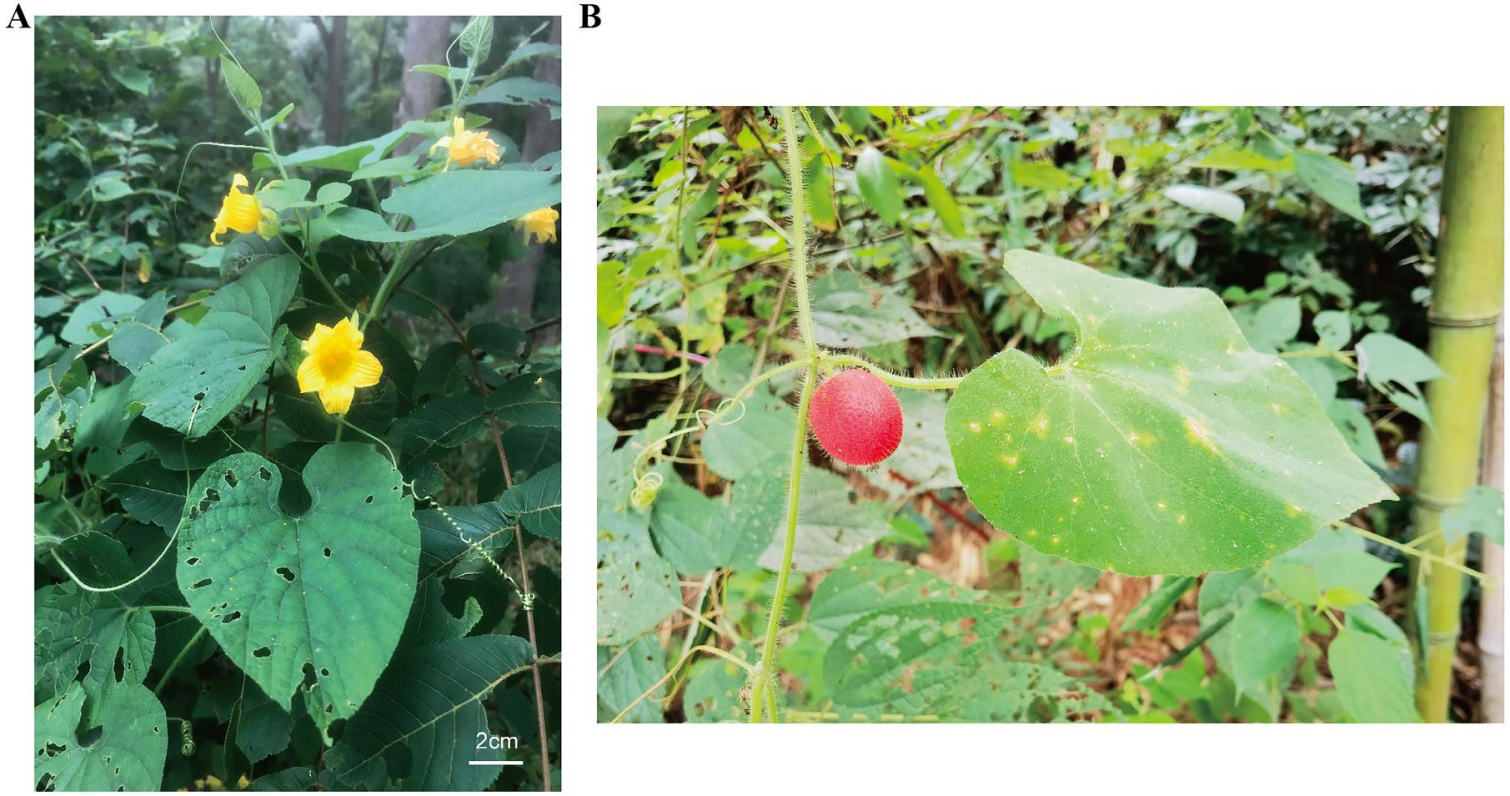
Species image of *Thladiantha nudiflora* showing the morphology of leaves and flowers (A) and fruit (B). Photograph was taken by Yan-Yan Zhao in Xuancheng, Anhui, China. Main identifying traits: Plants densely pubescent-hispid; leaves slightly stiff, blade ovate-cordate; with tendrils; female flowers solitary; flowering in spring and summer; fruiting pedicel robust, 2.5–5.5 cm; fruit red or red-brown when mature.

The leaves of *T. nudiflora* were gathered from Xuancheng, Anhui, China (GPS: 118°41′30.73″E 30°34′15.49″N). The samples were deposited at the herbarium of the College of Life Sciences and Medicine, Zhejiang Sci-Tech University, Hangzhou (Plant Genomics & Molecular Improvement of Colored Fiber Lab, http://sky.zstu.edu.cn/, Identifier: Yan-Yan Zhao, zhaoyanyanhao@zstu.edu.cn) under voucher number ZSTUTN01. The leaves and flowers of *T. nudiflora* were photographed by ourselves and shown in Figure 1. No permits are needed to get the sample because this species is neither endangered nor protected.

Using the DNA Plantzol Reagent (Invitrogen, Carlsbad, CA, USA), total genomic DNA was extracted from leaves according to the manufacturer’s recommendations. Sequencing libraries were procured as directed by the manufacturer using Illumina’s TruSeq Nano DNA Library Preparation kit (350 bp median insert). The library was sequenced on the Illumina HiSeq 2500 platform (Illumina Inc., San Diego, CA, USA). All raw readings were processed with Trimmomatic v0.39 software (Jülich, Germany) to remove adaptor sequences, short reads (length 75 bp), and low-quality bases (*Q*-value 20). Then, with adaptors trimmed, 21.3 million high-quality raw reads (150 bp paired-end read length) were produced. The plastid-like readings were acquired from clean reads and assembled using the GetOrganelle ver.1.7.6.1 (Jin et al. 2020). We further mapped our clean reads back to the assembled chloroplast genome to evaluate the depth of coverage in order to clarify the accuracy of the assembly (Supplementary Figure S1). The genome was annotated using Geneious v11.1.5 (Kearse et al. 2012), with the *Thladiantha dubia* chloroplast genome as a reference (GenBank: NC_046855). Manual corrections were made to annotation mistakes. The CPGview-RSG (http://www.1kmpg.cn/cpgview/) was used to illustrate the structural properties of the chloroplast genomes.

We received 17 available complete chloroplast genomes of Cucurbitaceae from the National Center for Biotechnology information database to corroborate the phylogenetic position of *T. nudiflora*. Using MAFFT v7.3 (Katoh and Standley 2013), the entire chloroplast genome sequence was aligned. Phylogenetic tree was constructed based on the complete chloroplast genome using maximum-likelihood (ML) and Bayesian inference (BI). ML phylogenetic tree was constructed using IQ-TREE v1.6.12 (Nguyen et al. 2015). The best-fitting model was determined by ModelFinder (Kalyaanamoorthy et al. 2017) and was GTR + F + R3 and the branch support was tested with 1000 replications. BI phylogeny was inferred using MrBayes3.2.7 (Ronquist et al. 2012) with the optimal model GTR + G + I. The analysis was run with the Markov chain Monte Carlo (MCMC) of 1 million generations, in which every 1000 generations were sampled and the first 25% of MCMC samples were discarded as burn-in.

## Results

**Figure 2.**
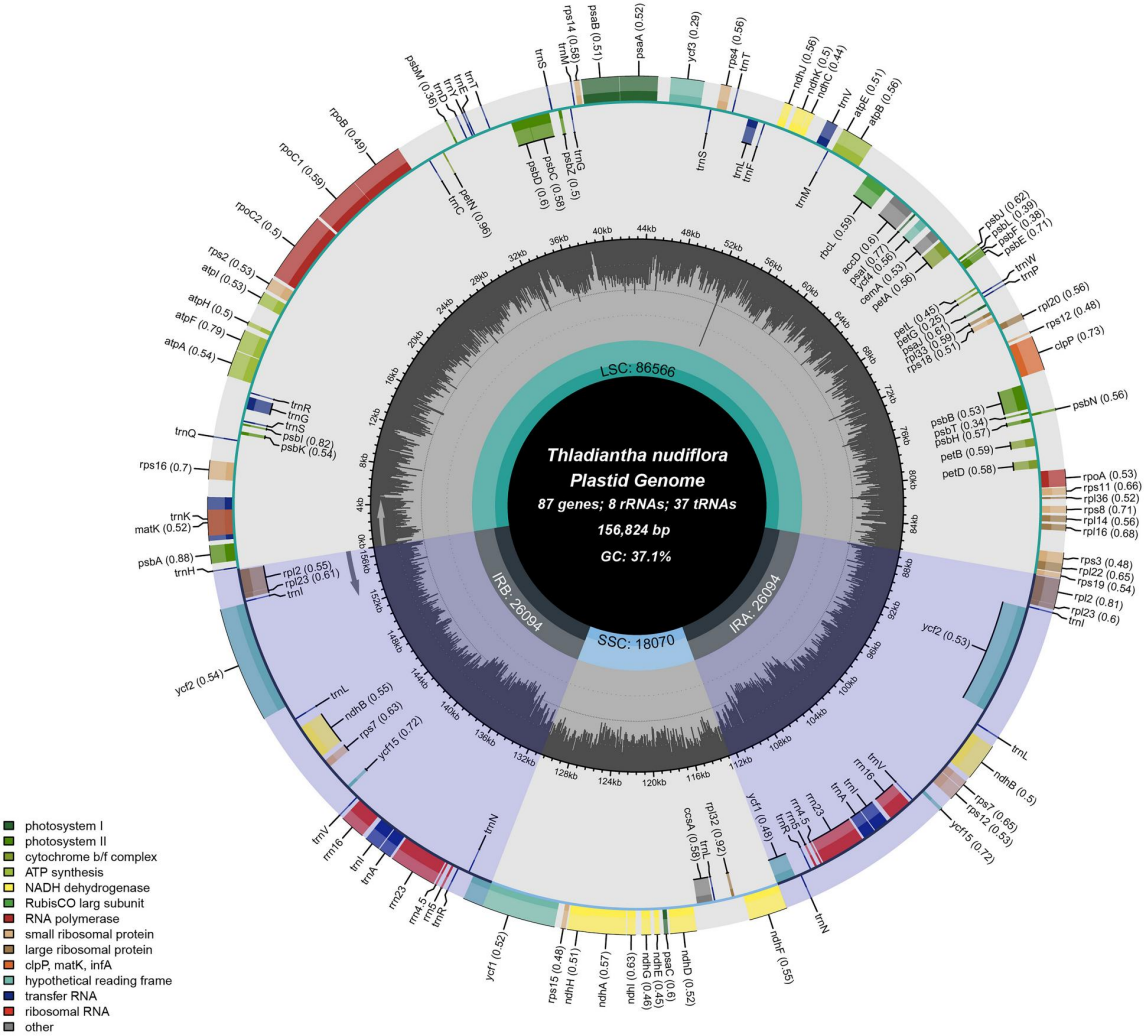
The entire map of *Thladiantha nudiflora*’s chloroplasts. The genes in the circle are transcribed clockwise while those on the outside are transcribed counterclockwise. Genes are colored differently according to their role. In the middle circle, the GC information is displayed in a deeper shade of gray, while the AT content is displayed in a lighter shade.

The full length of *T. nudiflora* chloroplast genome sequence (GenBank accession number OQ286031) was 156,824 base pairs and contained two inverted repeat (IR, 26,094 bp) sections, a large single-copy region (LSC, 86,566 bp), and a small single-copy region (SSC, 18,070 bp). The overall GC content was 37.1%, and the GC content of the IR, LSC, and SSC was 42.7%, 34.9%, and 31.2%, respectively. There were 132 genes in the genome (87 protein-coding, eight rRNA, and 37 tRNA) (Figure 2). Nineteen of these genes had another copy, including eight protein-coding genes (*rps7*, *ndhB*, *ycf2*, *rpl2*, *rps12*, *rpl23*, *ycf15*, *ycf1*), seven tRNA genes (*trnA-UGC*, *trnE-UUC*, *trnR-ACG*, *trnV-GAC*, *trnN-GUU*, *trnM-CAU*, *trnL-CAA*), and all four rRNA genes (*rrn5, rrn16, rrn4.5, rrn23*). Two introns were identified in three genes (*clpP*, *rps12*, *ycf3*) and one intron in five protein-coding genes (*rpl2*, *ndhB*, *rps16*, *rpoC1*, *ndhA*) (Supplementary Figure S2).

**Figure 3.**
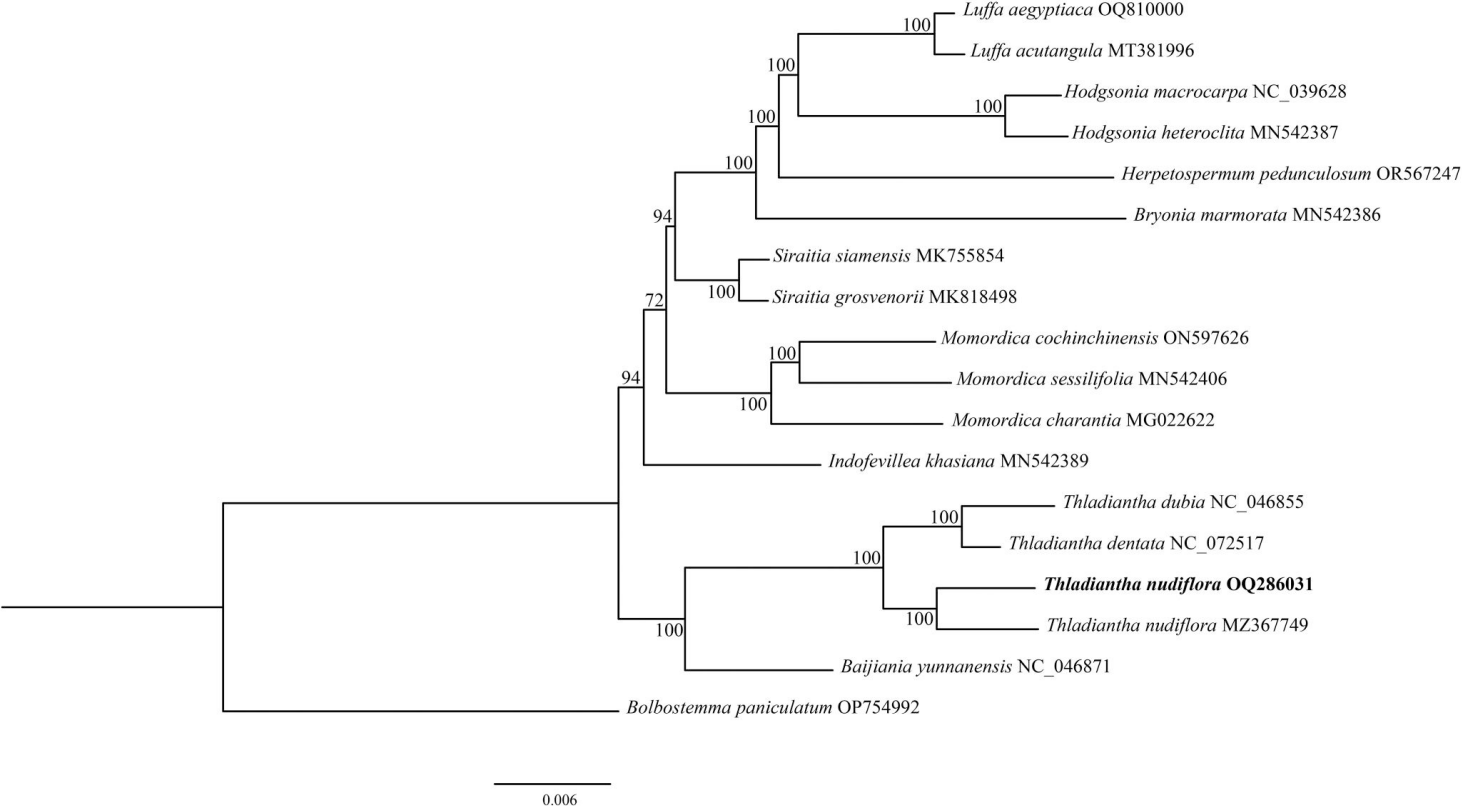
Phylogenetic tree of 18 species in Cucurbitaceae was constructed based on the complete chloroplast genome using maximum-likelihood (ML). Numbers at the nodes indicate bootstrap support values, and the scale bar represents nucleotide substitutions per site. *Bolbostemma paniculatum* was used as outgroups. The following sequences were used: *Baijiania yunnanensis* (NC_046871, Bellot et al. 2020), *Bolbostemma paniculatum* (OP754992), *Bryonia marmorata* (MN542386, Bellot et al. 2020), *Herpetospermum pedunculosum* (OR567247, Wang et al. 2020), *Hodgsonia heteroclita* (MN542387, Bellot et al. 2020), *Hodgsonia macrocarpa* (NC_039628, Zeng et al. 2018), *Indofevillea khasiana* (MN542389, Bellot et al. 2020), *Luffa acutangula* (MT381996, Yundaeng et al. 2020), *Luffa aegyptiaca* (OQ810000), *Momordica charantia* (MG022622), *Momordica cochinchinensis* (ON597626, Cai et al. 2023), *Momordica sessilifolia* (MN542406, Bellot et al. 2020), *Siraitia grosvenorii* (MK818498), *Siraitia siamensis* (MK755854, Shi et al. 2019), *T. dentata* (NC_072517), *T. dubia* (NC_046855, Bellot et al. 2020), *T. nudiflora* (MZ367749), *T. nudiflora* (QQ286031).

To understand the phylogenetic position of *T. nudiflora* in Cucurbitaceae, we downloaded complete chloroplast genomes of 17 species of the Cucurbitaceae family from the NCBI GenBank database. Most cucurbits have berry or pepo fruits (Barrera-Redondo et al. 2020). Since *Bolbostemma paniculatum* is one of the few capsule fruits in Cucurbitaceae, it was used as an outgroup to construct the phylogenetic tree. The phylogenies reconstructed by ML methods (Figure 3) and BI (Supplementary Figure S3) were topologically identical. Species of the genus *Thladiantha* were clustered together in the phylogenetic trees, suggesting this genus was a monophyletic group. Additionally, *Baijiania yunnanensis* exhibited as the sister clade to the species of the genus *Thladiantha*.

## Discussion and conclusion

At present, there is little research on the phylogeny of *Thladiantha*, mainly because there are few chloroplast genomes published. In this study, the chloroplast genome sequence of *T. nudiflora* was assembled and annotated, which can be subsequently used for DNA barcoding and molecular phylogeny of *Thladiantha*. The complete chloroplast genome of *T. dubia* and *Thladiantha dentata*, other species of the genus *Thladiantha*, have been publicly available (Bellot et al. 2020). Phylogenetic analysis showed that species of the genus *Thladiantha* were clustered together in the phylogenetic trees, suggesting this genus was a monophyletic group. Additionally, *Baijiania yunnanensis* exhibited as the sister clade to the species of the genus *Thladiantha*. In conclusion, this study will not only shed light on *T. nudiflora*’s evolutionary position but also provide valuable chloroplast genomic information for future studies into the origins and diversification of the genus *Thladiantha* and the Cucurbitaceae family.

## Supplementary Material

Supplemental Material

Supplemental Material

Supplemental Material

## Data Availability

The genome sequence data that support the findings of this study are openly available in GenBank of NCBI (https://www.ncbi.nlm.nih.gov) under accession no. OQ286031. The associated BioProject, SRA, and Bio-Sample numbers are PRJNA822536, SRR18578198, and SAMN27191640, respectively.

## Health and safety

The authors certify that any disclosed experimental work was carried out in accordance with the necessary laboratory health and safety protocols.
