# Supplementary figures and images for: The complete chloroplast genome sequence of Thladiantha nudiflora Hemsl. ex F.B.Forbes & Hemsl. 1887 (Cucurbitaceae)
Source: Mitochondrial DNA B Resour. 2024 Jan 25;9(1):138–42. doi: 10.1080/23802359.2024.2305402 (PMC10812858; doi:10.1080/23802359.2024.2305402)

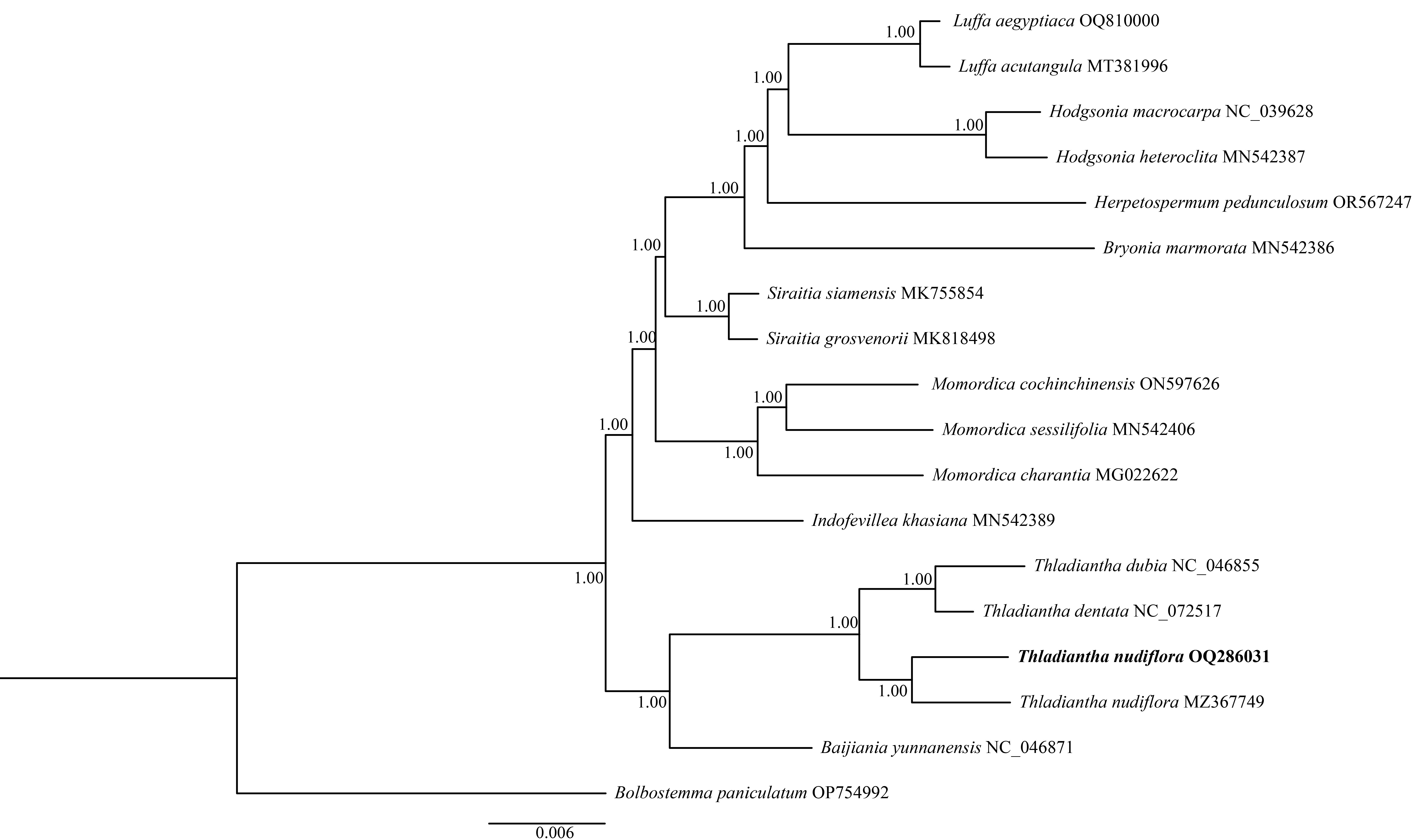

Supplement: Supplemental Material [file TMDN_A_2305402_SM7095.jpg]

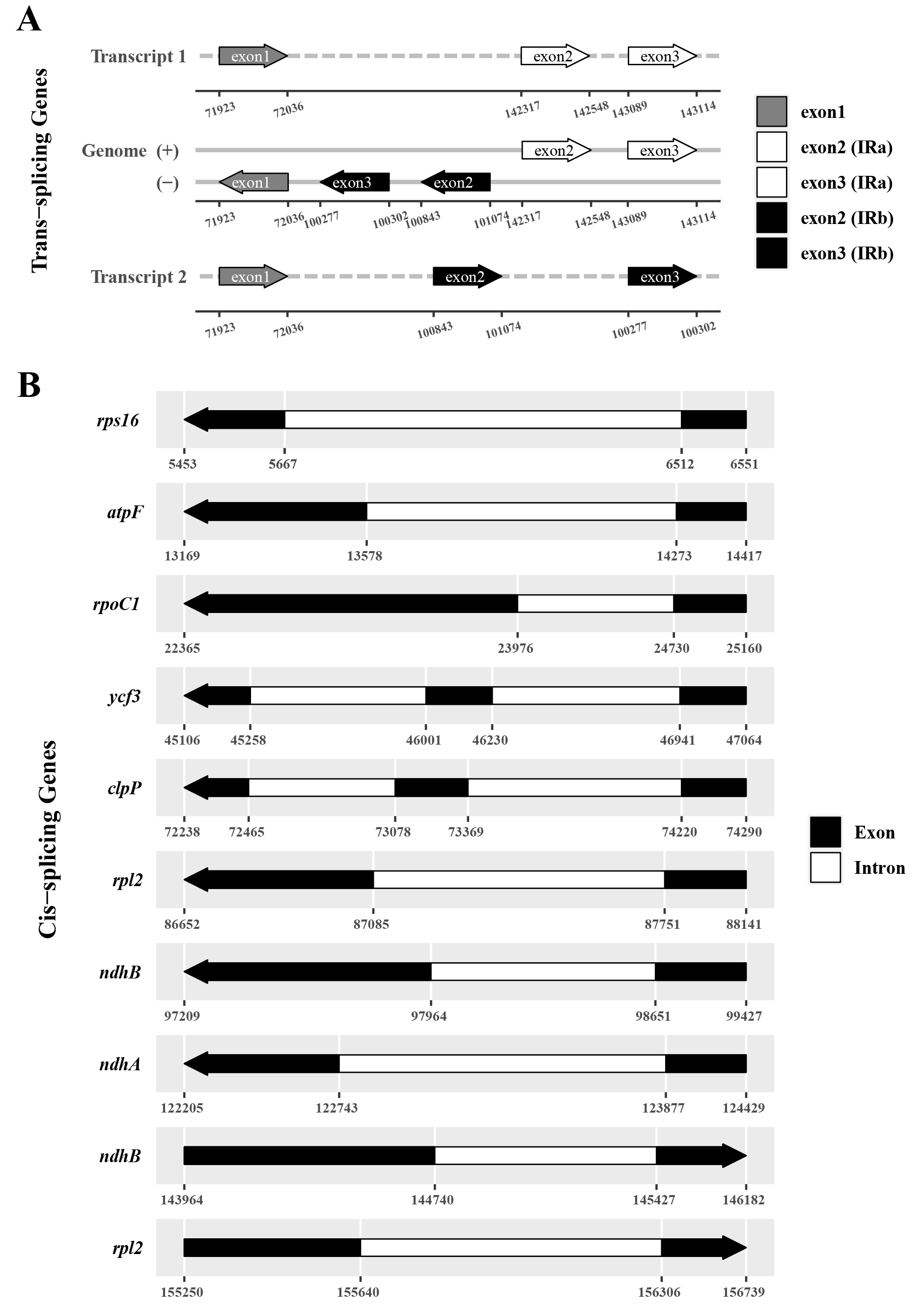

Supplement: Supplemental Material [file TMDN_A_2305402_SM7092.jpg]

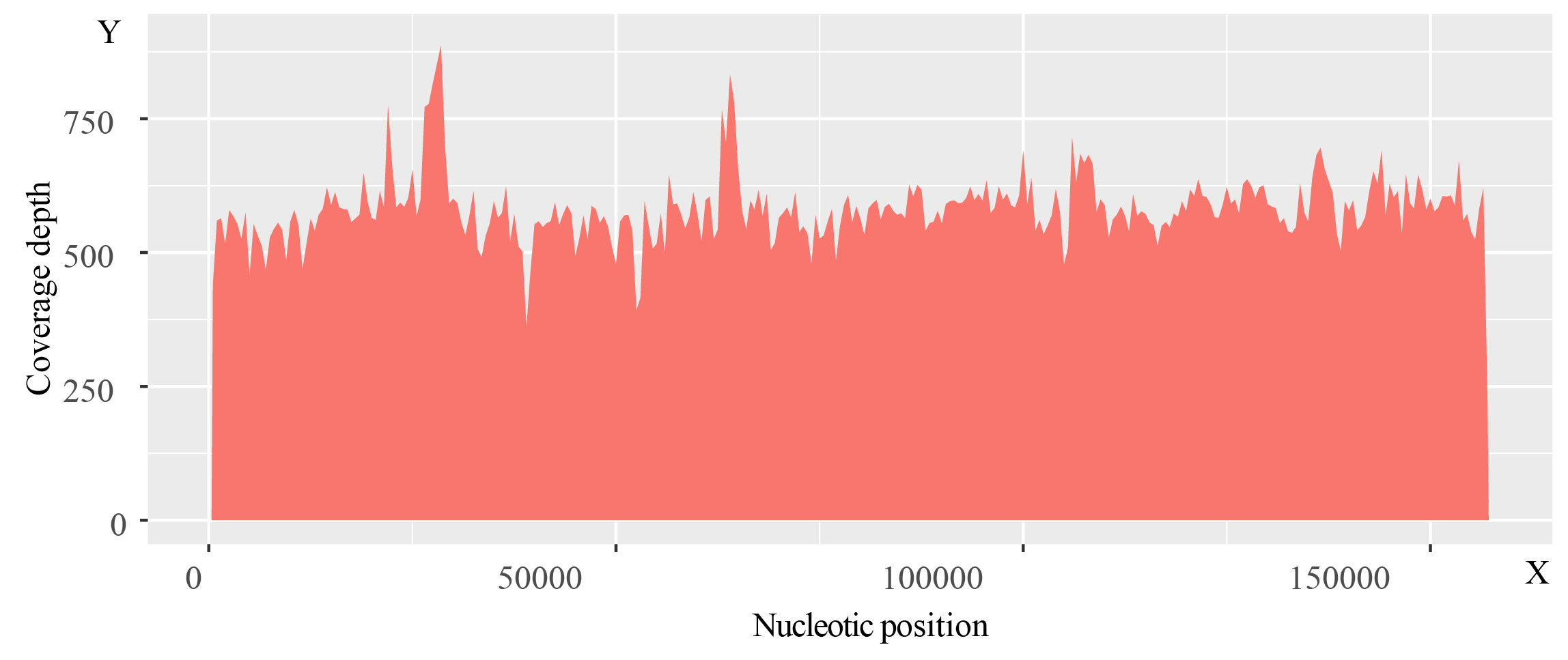

Supplement: Supplemental Material [file TMDN_A_2305402_SM7091.jpg]
